# Cotton rat lung transcriptome reveals host immune response to Respiratory Syncytial Virus infection

**DOI:** 10.1038/s41598-018-29374-x

**Published:** 2018-07-27

**Authors:** Seesandra V. Rajagopala, Harinder Singh, Mira C. Patel, Wei Wang, Yi Tan, Meghan H. Shilts, Tina V. Hartert, Marina S. Boukhvalova, Jorge C. G. Blanco, Suman R. Das

**Affiliations:** 1 Department of Medicine, Vanderbilt University Medical Center, Nashville, Tennessee USA; 2 Infectious Diseases Group, J. Craig Venter Institute, Rockville, Maryland USA; 3 Sigmovir Biosystems Inc, Rockville, Maryland USA

## Abstract

Acute respiratory infection (ARI) with respiratory syncytial virus (RSV) is the most common cause of both hospitalizations and mortality in young infants worldwide. Repeat infections with RSV are common throughout life in both pediatric and elderly populations. Thus far, cotton rats (*Sigmodon hispidus*) are found to be the best animal model to study RSV infection. However, the lack of a cotton rat reference genome limits genome-wide host gene expression studies. We constructed the first lung tissue *de novo* transcriptome for the cotton rat. Cotton rat lung tissue transcripts were assigned to 12,211 unique UniProt genes, which were then utilized to profile the host immune response after RSV infection. Differential expression analysis showed up-regulation of host genes involved in cellular functions including defense responses to viral infection and immune system processes. A number of transcripts were downregulated during the later stage of infection. A set of transcripts unique to RSV-infected cotton rats was identified. To validate RNA-Seq data of three such transcripts (TR453762, TR529629, and TR5333), their expression was confirmed by quantitative real-time polymerase chain reaction.

## Introduction

Human respiratory syncytial virus (RSV), a member of the paramyxovirus family, is a leading cause of severe acute lower respiratory tract infection in infants, the elderly, and immunocompromised adults. There are no effective vaccines or treatments available yet; however, ~60 RSV vaccines candidates are currently in development^1^. So far, a complete integrated picture of RSV-host interaction is still lacking^2,3^. Animal models are critical in understanding viral replication and pathogenesis *in vivo* and essential for developing countermeasures against viral infections. The cotton rat (*Sigmodon hispidus*) is a small rodent susceptible to a large variety of human pathogens^4^. This model is best known for its use in research related to respiratory viruses^5^ and is a well-established model for RSV infection^4^. Previous studies have shown that permissiveness of the cotton rat to infection with human RSV exceeds that of mice by more than 100-fold^4^. Molecular level analysis reveals important features of the model that make the cotton rat stand out in comparison to other small animal models. For example, the cotton rat carries a functional set of Mx genes encoding antiviral proteins Mx1 and Mx2^6^. Human Mx proteins are crucial components of the innate antiviral defense system, which, together with adaptive immune mechanisms, facilitates clearance of viral infections^4^. In contrast, most common laboratory strains of mice lack a functional Mx system, and murine antiviral defense relies mostly on an incomplete type I interferon response adaptive immune mechanisms^6^. As of today, the reference genome for the cotton rat does not exist in the public domain, which limits studies to characterize host response on a global scale; for example, studies of host gene expression after viral infection.

Next-generation sequencing techniques facilitate rapid generation of transcriptome assemblies for any species of interest^7^. Recent advances in RNA sequencing (RNA-Seq) technology and *de novo* transcriptome assembly tools enable large-scale analysis of transcriptomes in an organism without the need for whole genome sequencing. A comprehensive gene space for a species can be generated using RNA-Seq and *de novo* transcriptome assembly platforms, which facilitates rapid discovery of novel genes and gene expression profiles for the specific biological conditions. Moreover, RNA-Seq followed by d*e novo* transcriptome analysis provides an excellent platform to generate a comprehensive resource of the gene space in an organism without whole genome sequencing and allows rapid and accurate quantification of transcript abundance in a given biological sample^8–11^. Given the importance of the cotton rat as a useful animal model in understanding the pathogenesis of RSV and other respiratory viral infections, we aimed to sequence, assemble, and annotate a lung transcriptome, as lung is the active site for RSV infection and replication. The *de novo* assembled, annotated transcriptome sequences provide an invaluable resource for the identification of cotton rat genes involved in viral replication and pathogenesis. Furthermore, the assembled transcriptome was used to investigate the host transcriptional response to RSV infection. The gene expression profiles upon RSV infection provide an invaluable resource for the identification of essential pathways involved in the antiviral defense system, adaptive immune mechanisms, and the resolution of infections.

## Results

### Study design

**Figure 1:**
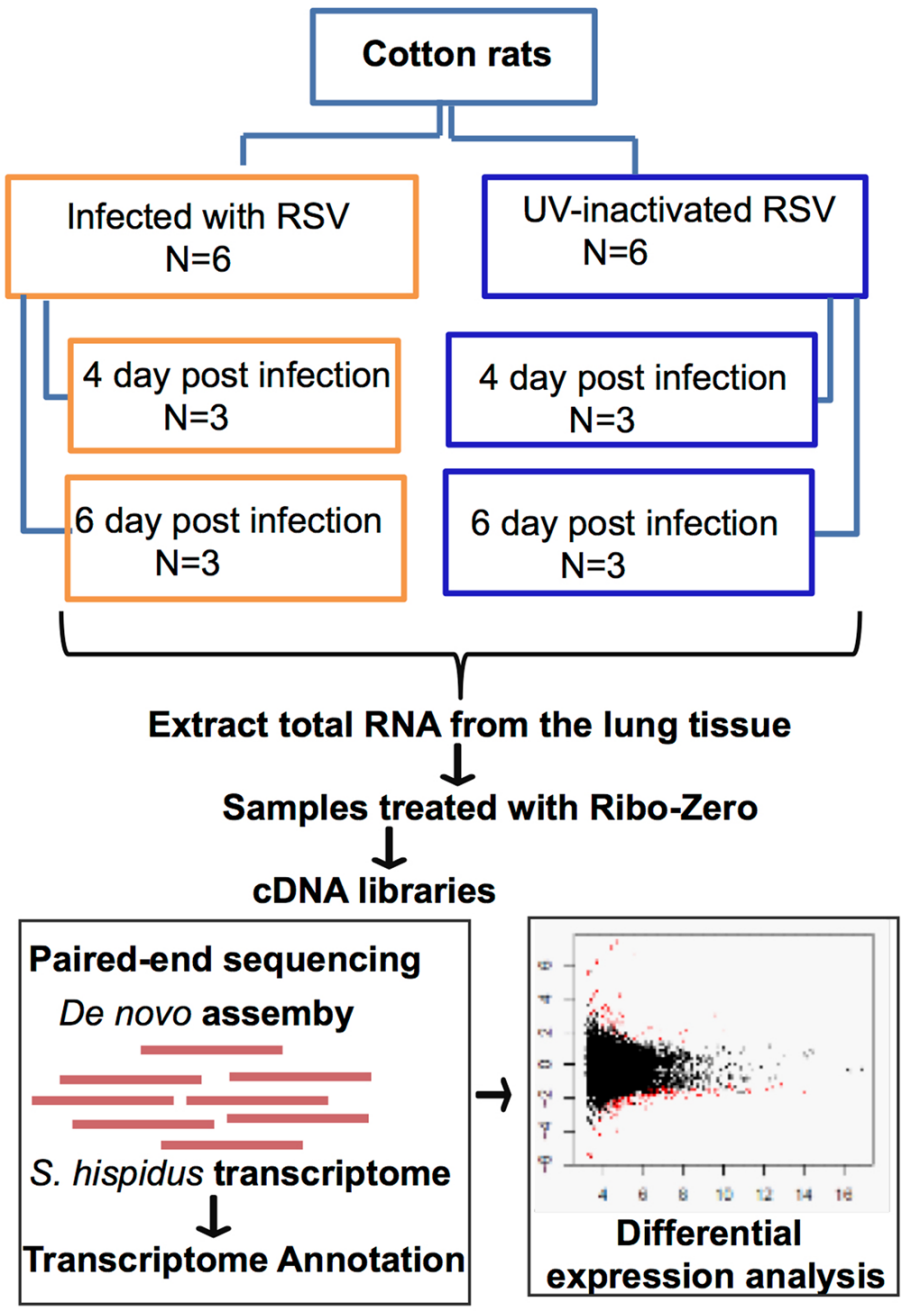
Flowchart outlining the study design. Cotton rat lung tissue was used to construct the lung transcriptome. Cotton rats infected with RSV and mock-infected (UV-inactivated virus) animals were used to assess the host response to RSV infection at the transcriptome level at day 4 and 6. The transcripts from both mock-infected and RSV-infected animal’s lung tissue samples were used for transcriptome assembly and annotation.

We used the inbred cotton rat to build a reference transcriptome and to determine the host response to RSV infection at the transcript level. A total of 12 young female cotton rats (4–6 weeks old, ~100 g) were used in this study. The experimental group (6 rats; 3 rats/cage) was intranasally (i.n.) infected with live RSV [RSV/A/Long (10^5^ pfu in 100 μl)], while the control group (6 rats; 3 rats/cage) was inoculated i.n. with UV-inactivated RSV (equivalent to 10^5^ pfu in 100 μl). Three animals from each group were sacrificed at days 4 and 6 post-infection. Total RNA isolated from the lung left lobe was used to genarate the cDNA libraries. Paired-end sequencing with 2 × 150 nt reads was performed on the Illumina NextSeq500 instrument. After quality trimming, the paired-end reads from the lung tissue from both, control inoculated (UV-inactivated RSV) and RSV-infected animal were used for *de novo* assembly. The *de novo* assembled transcripts were annotated using Trinotate pipeline (https://trinotate.github.io/). The *de novo* assembled cotton rat transcriptome was then used as a reference database for the differential expression analysis at 4 and 6 days post infection (p.i) and to evaluate the host response to active RSV infection (Fig. 1).

### Cotton rat transcriptome sequencing and *de novo* assembly

Over 150 million paired-end (2 × 150 nt) raw reads were generated from the lung tissue transcripts, which approximately amounts to 110 GB transcriptome data (see methods). Low quality reads and reads with <80 bp in length were removed using Trimmomatic^12^. From the remaining high-quality paired-ends reads, we sampled ~75 million reads for the *de novo* transcriptome assembly. The Trinity pipeline with *in silico* normalization approach was used for *de novo* transcriptome assembly^7^ (see methods). The Trinity assembler generated a total of 771,188 contigs with 443,491,781 bases (N50 length, 657 bp; median contig length, 365 bp) and a GC% of 43.22%. These cotton rat transcripts generated by the *de novo* assembly were designated as putative genes or gene isoforms, which were assigned a unique identifier. Before subjecting the *de novo* assembled transcripts for annotation, the low-confidence transcripts were removed using FPKM (Fragments Per Kilobase of transcript per Million mapped reads) cut-off of 1. We used the RSEM software package^13^ for quantifying transcripts abundance (see Methods). About 135,805 assembled transcripts were retained after FPKM > 1 cut-off; these transcripts were subjected to functional annotation (Table 1).

**Table 1:** Cotton rat transcriptome assembly and annotation.

**Transcriptome assembly**	
Total trinity transcripts	771,188
Total trinity transcripts (FPKM > 1)	135,805
Percent GC	43.22
Contig N50	657
Median contig length	365 bp
Average contig length	575.08 bp
Total assembled bases	443,491,781 bp
**Annotation**	
Functionally annotated transcripts	38,736
Unique genes (UniProt)	12,211

### Functional annotation of cotton rat transcripts

**Figure 2:**
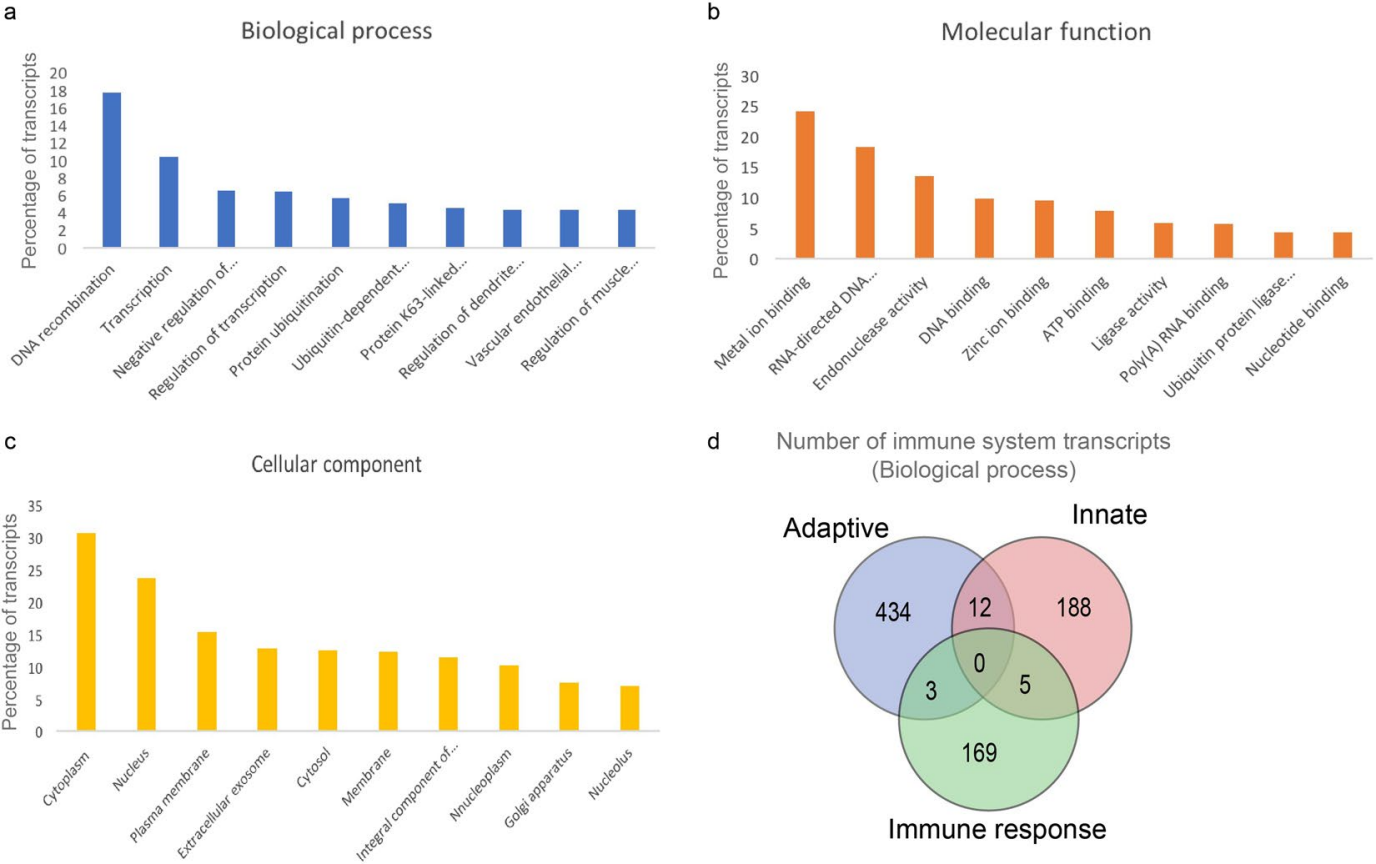
Gene Ontology (GO) categories of RSV transcripts. Distribution of the GO categories assigned to the RSV transcriptome. The x-axis represents the name of the GO categories, and the y-axis represents the ratio of the number of annotated transcripts for each GO to the total number of the annotated transcripts. (**a**) Annotated transcripts in the biological process, (**b**) cellular component and (**c**) molecular function categories. (**d**) Venn diagram showing the number of adaptive immune response, innate immune and immune response transcripts in the cotton rat lung transcriptome.

In the absence of an annotated genome, functional predictions for the *de novo* assembled transcripts are essential to understand the diverse molecular functions, biological processes, and unique pathways represented in an organism. Cotton rat transcripts were annotated based on sequence homology with protein domains in a manually curated UniProt protein database using BLASTx against the UniProt protein database^14^. This sequence homology search works by aligning *de novo* assembled cotton rat transcripts with a non-redundant protein database (UniProt), then annotating them based on sequence similarity. Out of 135,805 *de novo* assembled cotton rat lung transcripts, 38,736 transcripts were functionally annotated with high confidence (e-value ≤ 1e − 05) (Table S1) and these transcripts were assigned to 12,211 unique (Uniprot) genes (Table S2). In addition to being annotated for Gene Ontology (GO) terms, the transcripts were screened for open reading frames (ORFs), to predict the amino acid sequence of proteins derived from these transcripts. The entire transcriptome annotation details can be found in Supplementary Table S1. The transcriptome functional annotation, GO terms were assigned to 11,934 (98.5%) of the annotated genes (Table S2)^15^. Under biological process GO term annotation; the top GO terms were DNA recombination 2,161 (17.7%), transcription 1,259 (10.3%) and regulation of transcription 785 (6.5%), and protein ubiquitination 687 (5.6%) (Fig. 2a). The most common molecular function GO terms were metal ion binding 2,938 (24%), RNA-directed DNA polymerase activity 2,234 (18.3%), endonuclease activity 1,649 (13.5%), DNA binding 1,206 (10%), and zinc ion binding 1,159 (9.5%) (Fig. 2b). The most cellular component GO terms were cytoplasm (30.7%), nucleus (15%), plasma membrane (15%), and extracellular exosome (13%). A complete list of transcripts GO annotations is available in Supplementary Tables S3 and S7. The cotton rat transcripts that were assigned to 12,211 UniProt genes includes 811 transcripts that are annotated to immune system functions. These transcripts were further subclassified as to adaptive immune system (449), to the innate immune system (205) and to immune response (177) functions (Fig. 2d).

### Pathway analysis to identify homologous pathways in cotton rat

**Figure 3:**
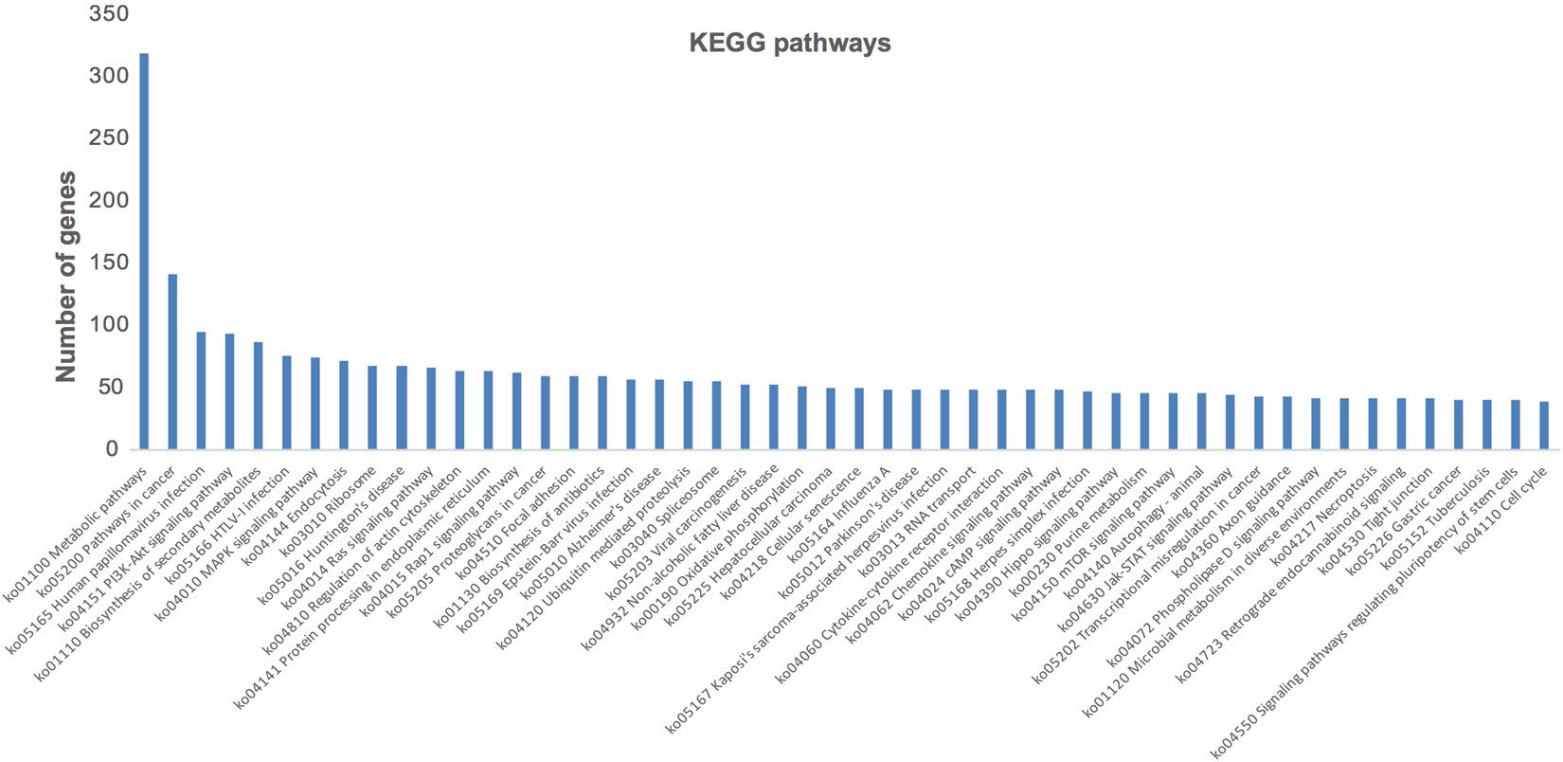
KEGG pathway analysis. The x-axis represents the name of the KEGG pathway and the y-axis represents the total number of genes present in the cotton rat transcriptome, which are part of that KEGG pathway^18^ Data is shown for top 50 KEGG pathway (www.kegg.jp/kegg/kegg1.html).

**Figure 4:**
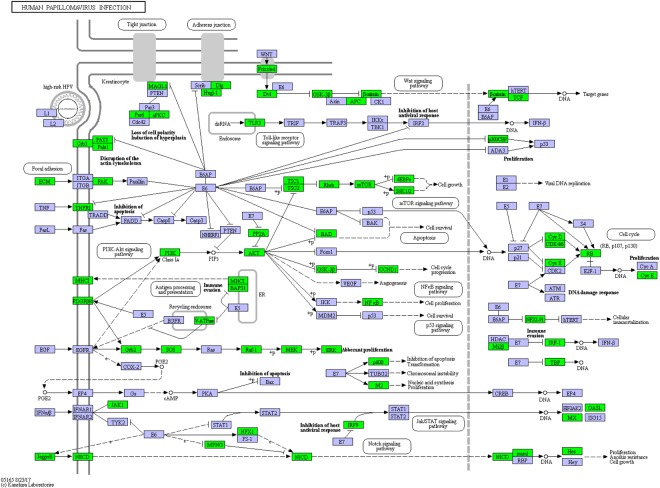
Mapping transcripts on KEGG pathway. The cotton rat transcriptome was mapped to KEGG reference human papillomavirus infection pathway (image reproduced with permission from KEGG)^18^ components with homologues in the cotton rat are colored in green (boxes).

The Kyoto Encyclopedia of Genes and Genomes (KEGG) enables visualization of metabolic pathways and molecular interaction networks captured in the *de novo* transcriptome^16^. The cotton rat is a well-established model organism to study respiratory infectious diseases, especially RSV. The cotton rat has been used as an animal model to evaluate novel therapies for RSV because it has shown a similar infection and disease pattern as humans and has an established translational value^4,17^. A total of 8,579 transcripts were annotated to a KEGG Orthology (KO) group (Table S1). The KEGG Mapper tools available on KEGG website were used to map the annotated transcripts against all the pathways available in KEGG. The annotated transcripts (at the gene level) were mapped to pathways in the KEGG database. The KEGG analysis showed that 8,579 transcripts were assigned to 369 pathways. The top 50 KEGG pathways are shown in Fig. 3, and the most highly expressed pathways were “metabolic pathways” (total of 319 transcripts) followed by “pathways in cancer” (141), “pathways of human papillomavirus infection” (95), and “pathways during Human T-Cell Lymphotropic Viruses Type-I (HTLV-I) infection” (76). We investigated the presence of innate and adaptive immune system genes in the *de novo* assembled cotton rat transcriptome by mapping the transcripts to pathways in the KEGG database^16,18^. The cotton rat’s lung transcriptome covered a total of 449 adaptive immune response, 205 innate immune response, and 177 immune response transcripts (Fig. 2d). The cotton rat transcriptome contains 49 genes for cytokine-cytokine receptor interaction, 29 genes for TNF-signaling, and 26 genes for Toll-like receptor pathway (Table S4). The KEGG reference pathway mapping revealed that most of the components of the KEGG human papillomavirus infection pathway were present in our annotated cotton rat transcriptome (Fig. 4).

### Differential gene expression analysis

**Figure 5:**
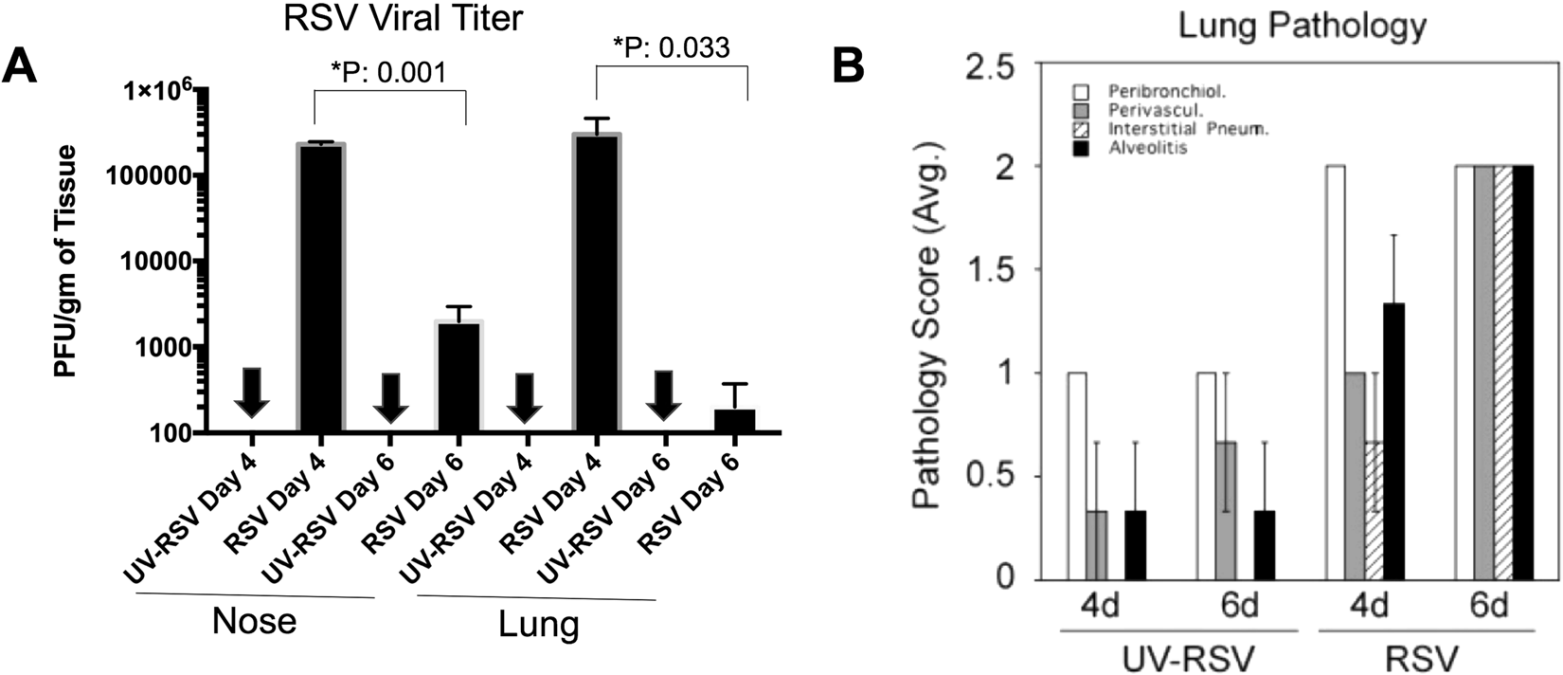
(**A**) RSV viral titers and (**B**) lung pathology determined for the animals. Animals were infected with RSV (10^5^ PFU/animal) or inoculated with the same dose of a UV inactivated virus (UV-RSV). Animals were sacrificed at the indicated days p.i. Lung and nose homogenates were used to measure virus load and the right lobe of the lung inflated with formalin and H&E stained for histological analysis. Each group represented by n = 3. Arrow mark scored below the limit of detection.

**Figure 6:**
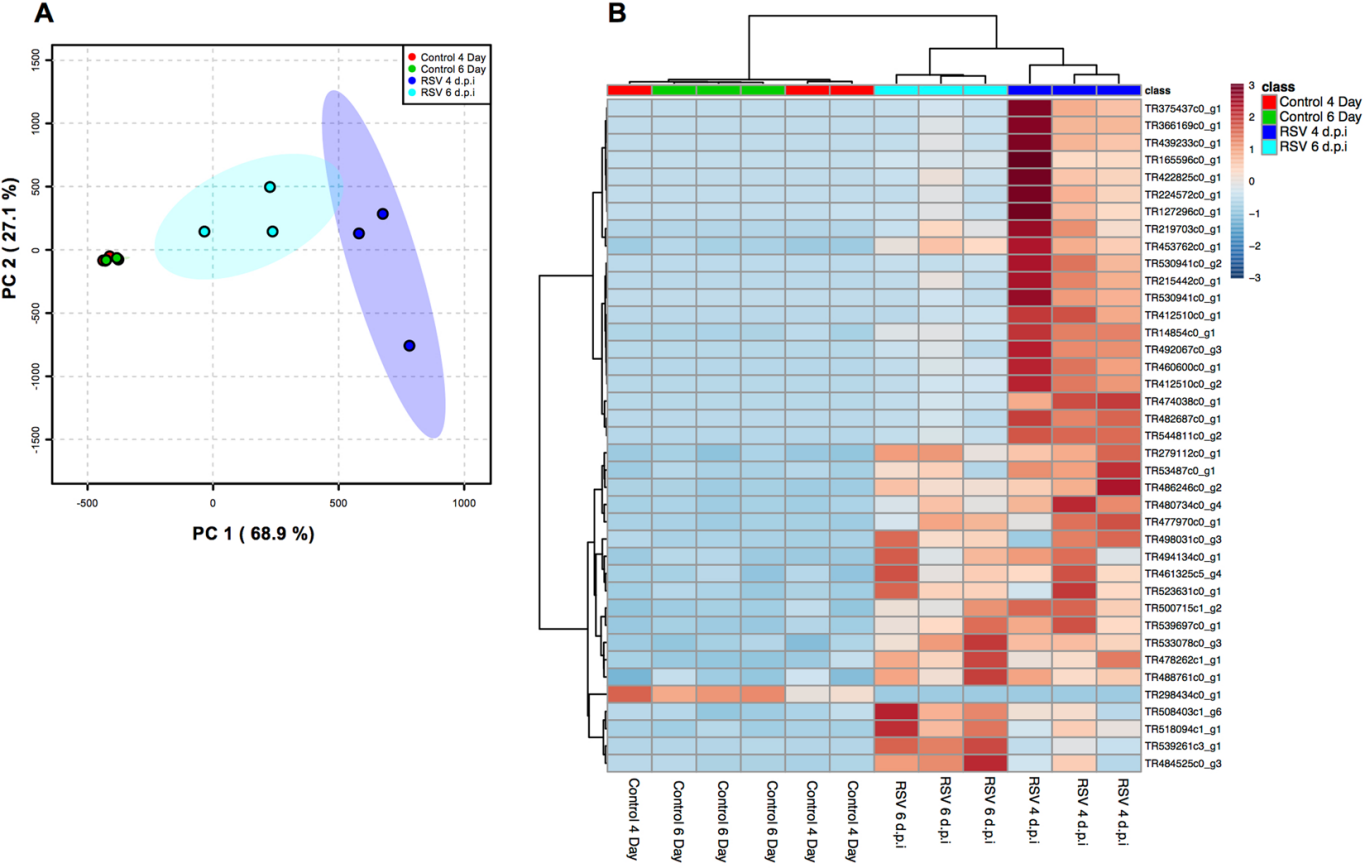
Cotton rat lung tissue gene expression cluster of the RSV-infected vs. mock-infected samples. (**A**) Principal component analysis (PCA) and (**B**) tree clustering (Euclidean) for the gene expression data. As expected the control sample at 4 days and 6 days p.i. grouping together based on the similarity of their gene expression patterns. The experimental sample at 4 days and 6 days p.i, were cluster distinct compare to control as well as at different time points of RSV infection.

**Figure 7:**
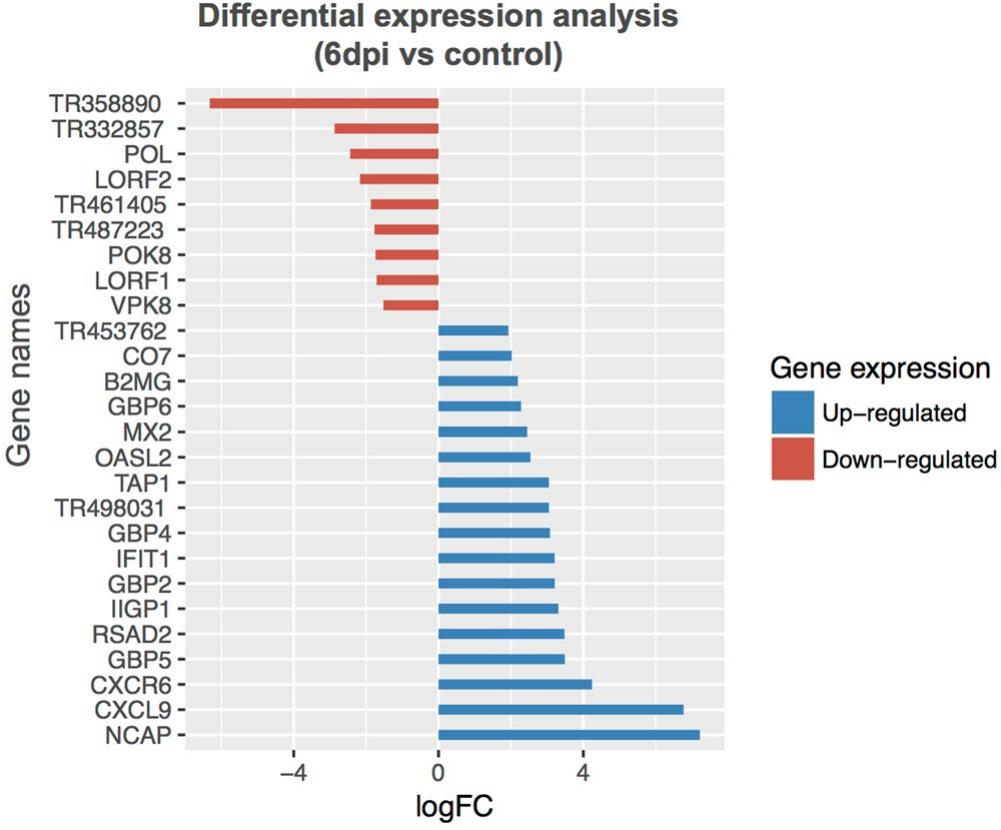
Gene expression changes upon RSV infection. The panel showing the genes that are differentially expressed in lung tissue between the control and RSV infected cotton rat at 6 days p.i.

RNA-Seq technology is a powerful approach for whole transcriptome sequencing, to measure gene expression profiles under different physiological conditions, and to profile tissue-specific gene expression^8^. We utilized the *de novo* assembled cotton rat transcripts as a reference to investigate host response to RSV infection. RSV viral replication in cotton rats infected i.n. was confirmed by determining RSV lung titers and lung histopathology (Fig. 5). As expected, peak RSV replication and viral gene expression in the lungs of cotton rats occurred on day 4 p.i. and anticipates the peak lung pathology that occurs later, on day 6 p.i.^19,20^. The RNA-Seq method was used to profile the host gene expression profiles after RSV infection. RNA-Seq data were obtained from lung tissues of 3 RSV infected cotton rat for each of the time points selected (4 days and 6 days p.i), and from 3 mock-infected cotton rats inoculated with UV inactivated RSV (UV-RSV) and sacrificed at the same time points. Transcripts were mapped to *de novo* assembled cotton rat transcripts as a reference database using RSEM^13^. Differential gene expression analysis was performed using the edgeR package^21^. In brief, for each sample, the raw transcripts counts were normalized using the TMM normalization method^13^. After normalization, the differential expression analysis was performed between the mock-infected and RSV-infected animals^21^. Gene expression values in each pair of replicates and control samples showed high correlation (Fig. 6). The gene expression analysis between the mock-infected group and the RSV-infected group at four days p.i. identified 69 cotton rat genes that were significantly up-regulated (FDR < 0.05) in response to infection (Tables 2 and S3). None of the cotton rat genes were significantly down-regulated at 4 days post RSV infection. At six days p.i., 67 genes were up-regulated, and 141 genes were down-regulated with an FDR of <0.05 (Fig. 7 and Table S4). In addition to host transcripts, RNA sequencing also captured viral transcripts as we used total RNA of the lung tissue to make the RNA-Seq libraries (see Methods section). As expected, most of the viral genes were predominant at 4 days p.i., (peak viral replication). Higher RSV titers were detected in lung and nose samples in the RSV 4 dpi compared to those in the RSV 6 dpi (Fig. 5), statistically significant. In contrast, lung pathology (mostly host inflammatory responses) appeared to be higher in the RSV 6 dpi. Changes in Perivasculitis and Intertitial pneumonia between RSV 4 and 6 p.i. are statistically significant by student T-test (p < 0.05).

**Table 2:** list of top 10 differentially regulated *cotton rat* genes.

Transcript	Homolog	LogFC	P Value
**4 days post infection**			
*TR453762|c0_g1		3.38	5.67E-11
TR480734|c0_g4	MX2_MOUSE: Interferon-induced GTP-binding protein Mx2	3.44	1.75E-09
*TR53487|c0_g1		3.81	4.05E-09
TR14854|c0_g1	OASL2_RAT: 2′-5′-oligoadenylate synthase-like protein 2	4.22	4.89E-09
TR279112|c0_g1	TAP1_RAT: Antigen peptide transporter 1	2.68	1.82E-08
*TR529629|c0_g1		3.56	2.56E-06
*TR486246|c0_g2		4.48	4.72E-06
*TR5333|c0_g1		3.73	2.09E-05
TR523631|c0_g1	IFIT1_MOUSE: Interferon-induced protein with tetratricopeptide repeats 1	3.38	4.14E-05
TR539697|c0_g1	RSAD2_RAT: Radical S-adenosyl methionine domain-containing protein 2	3.15	4.43E-05
**6 days post infection**			
TR539261|c3_g1	CXCL9_MOUSE: C-X-C motif chemokine 9	6.76	2.25E-20
*TR498031|c0_g3		3.05	2.21E-12
TR518094|c1_g1	GBP2_MOUSE: Guanylate-binding protein 1	3.21	1.28E-11
TR494628|c0_g2	GBP6_HUMAN: Guanylate-binding protein 6	2.27	8.69E-10
TR484525|c0_g3	GBP5_MOUSE: Guanylate-binding protein 5	3.49	9.92E-10
TR38018|c0_g1	CXCR6_MOUSE: C-X-C chemokine receptor type 6	4.23	4.92E-09
TR508403|c1_g6	GBP4_MOUSE: Guanylate-binding protein 4	3.08	9.54E-09
TR500729|c1_g7	TAP1_RAT: Antigen peptide transporter 1	3.05	5.39E-08
TR478262|c1_g1	IIGP1_MOUSE: Interferon-inducible GTPase 1	2.6	1.04E-07
*TR453762|c0_g1		1.92	1.58E-07

^*^No homolog found for the cotton rat transcript.

### Validation of differential gene expression data

**Figure 8:**
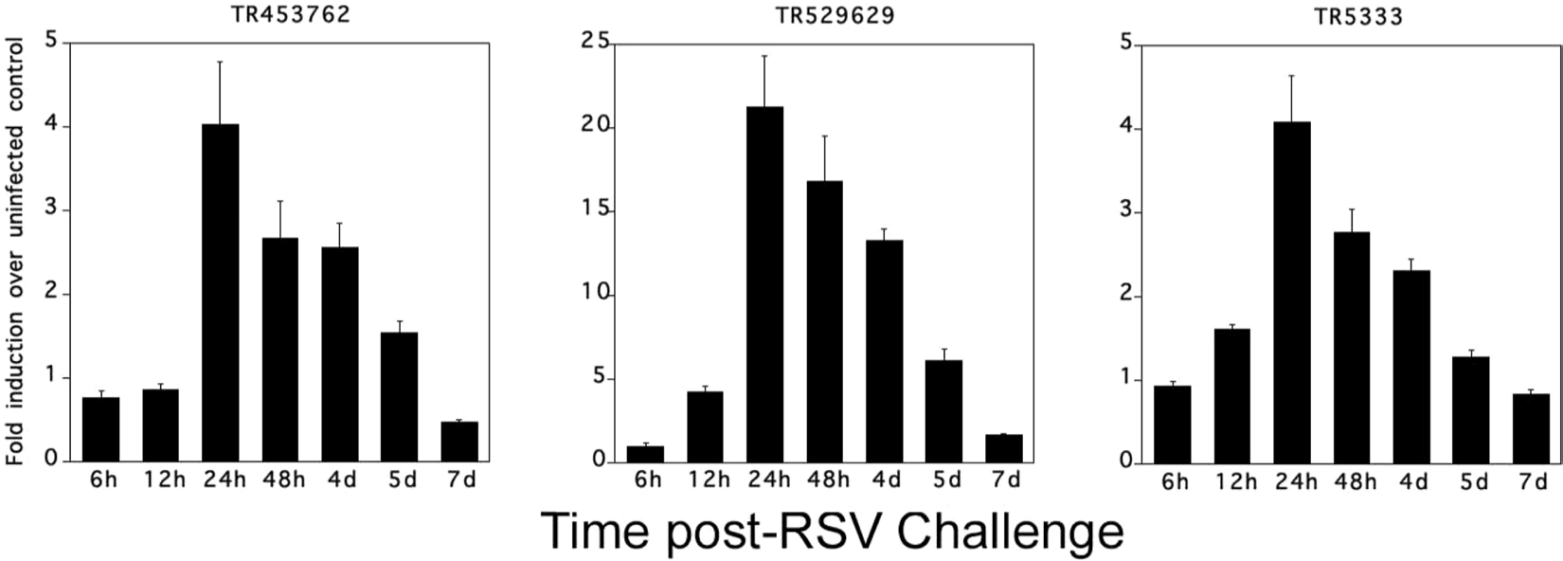
Validating the expression analysis data. We randomly selected three up-regulated transcripts, which were unique to cotton rat-based on RNA-Seq differential expression analysis. Real-time PCR primers were designed for each transcript. qRT-PCR measured relative mRNA expression profiles of the transcripts TR453762, TR529629, and TR5333 at 6 h, 12 h, 24 h, 48 h, 4d, 5d and 7d p.i. in the RSV-infected cotton rat lung tissues. The qRT-PCR results correlate with the RNA-Seq expression profiles.

To validate some of the transcripts, we selected three transcripts (TR453762, TR529629, and TR5333), which are unique to cotton rats, and were up-regulated based on RNA-Seq data at 4 days p.i. Real-time PCR primers were designed for each transcript. Using SYBR green,the quantitative real-time polymerase chain reaction (qRT-PCR) assays measured relative mRNA expression of the transcripts at 6, 12, 24, 48 hours and at 4, 5, and 7days post RSV-infection in lung using 4 animals per group. Gene expression level of these transcripts at different times post RSV infection correlates with the RNA-Seq data (Fig. 8).

### Gene ontology analysis of differential expression upon RSV infection

Those cotton rat transcripts that were differentially regulated after RSV infection were clustered according to their biological process, molecular function, and cellular component using GO databases. To define the major host biological processes and molecular functions altered upon RSV infection, we clustered the up-regulated cotton rat genes at 4 and 6 days p.i., using the REVIGO software^22^. As expected under biological process GOs, the assemblies of cellular component, the defense response, the innate immune response to the virus as well as the negative stranded viral RNA replication process are over-represented (Table 3). Likewise, under molecular function GOs, we found that large clusters of genes involved in various methyltransferase activity are top representative GO terms followed by GTP binding, and GTPase activity (Table 3).

**Table 3:** Gene Ontology terms enriched in differential expressed genes.

GO term	Description (Biological process)	Frequency	p-value
GO:0039689	negative stranded viral RNA replication	0.02%	0
GO:0060141	positive regulation of syncytium formation by virus	0.02%	0
GO:0051607	defense response to virus	0.97%	0
GO:0009615	response to virus	1.24%	0
GO:0045087	innate immune response	3.54%	0
	**Description (Molecular function)**		
GO:0004482	mRNA (guanine-N7-)-methyltransferase activity	0.01%	1.00E-300
GO:0005525	GTP binding	1.76%	0.001492451
GO:0005524	ATP binding	7.20%	7.36E-07
GO:0046979	TAP2 binding	0.01%	0.005344413
GO:0046978	TAP1 binding	0.01%	0.0087579
GO:0046980	tapasin binding	0.01%	0.00787771
GO:0045236	CXCR chemokine receptor binding	0.07%	0.016191982
GO:0048248	CXCR3 chemokine receptor binding	0.03%	0.012142273
GO:0008009	chemokine activity	0.20%	0.043491061
GO:0030332	cyclin binding	0.13%	0.046827395
GO:0042802	identical protein binding	6.73%	0.012752637
GO:0003924	GTPase activity	1.28%	8.15E-05
GO:0003725	double-stranded RNA binding	0.33%	0.006370888
GO:0003968	RNA-directed 5′-3′ RNA polymerase activity	0.01%	1.00E-300
GO:0001730	2′-5′-oligoadenylate synthetase activity	0.05%	4.21E-05
GO:0042288	MHC class I protein binding	0.07%	0.047217175
GO:0016814	hydrolase activity, acting on carbon-nitrogen (but not peptide) bonds, in cyclic amidines	0.13%	0.003102416
GO:0015433	peptide antigen-transporting ATPase activity	0.01%	0.00787771

## Discussion

The cotton rat is a small “new world” rodent species with an established history of being a model for virus research due to its susceptibility to a broad range of human viruses. The cotton rat is best known for its use in research related to respiratory viral infection, pathogenesis, and immunity^4,23^. Though the cotton rat model is ideal for studying mechanisms of respiratory virus pathogenesis and immunity, the reference genome for the cotton rat does not exist in the public domain, which limits studies to understand host response on a global scale. In this study, we constructed a *de novo* transcriptome of the cotton rat by assembling the high-quality paired-end reads generated through sequencing transcripts of cotton rat lung tissue; the transcripts were subsequently assembled using Trinity assembler^24^. About 16,383 cotton rat transcripts had significant homology with known mammalian genes, but a large number (~11,500) were novel transcripts that have yet to be characterized. Of transcripts of particular relevance for studies into pathogenesis and disease, we identified the presence of a series of known targets and functional pathways including the TNF signaling pathway, Toll-like receptor signaling, and human papillomavirus infection pathways that form part of their innate immune system. This *de novo* assembled transcriptome sequence and annotation data provides a significant molecular resource to study respiratory virus pathogenesis in cotton rats.

The *de novo* assembled cotton rat transcripts were used as a reference to investigate host response after RSV infection in cotton rat lungs after 4 and 6 days p.i. About 231 cotton rat genes were differentially expressed after RSV infection compared to the control group. Further, lung gene expression profiles were found to be different between RSV 4 dpi and RSV 6 dpi. Interstingly, similar numbers of host transcript differential expression were displayed in both RSV 4 dpi compared and the 6 dpi. Further, consistently higher RSV viral transcripts and the titers were detected in lung and nose samples in the RSV 4 dpi compared to those in the RSV 6 dpi. In contrast, lung pathology (mostly host inflammatory responses) appeared to be higher in the RSV 6 dpi (Fig. 5). We also found large number of transcripts (142) that are down modulated at day 6 compared to none for day 4. In past, we have shown that viral replication and RSV-induced gene expression preceded the bulk of inflammatory response in the lung of infected animals^19,20^. This is expected since there is a strong induction of inflammatory cytokines and chemokines, “the cytokine storm”, in the lung that results in the influx of inflammatory cells and lung damage. Most likely the strong interferon response early in infection is responsible of restraining the virus, but, as in many other infectious diseases, the process of healing and tissue remodeling takes longer and is correlated with the expression of a new set of genes an ddown modulation of the inflamatroy markers as evidenced on day 6.

For RNAseq validation, a subset of differentially expressed transcripts were further confirmed by qRT-PCR assays, which measured relative mRNA expression profiles of select transcripts at 6 h, 12 h, 24 h, 48 h, 4d, 5d and 7d in the RSV-infected cotton rat lung tissues (Fig. 8). The expression levels of transcripts were shown to be the highest at 24 h dpi, and then to progressively decline in all 3 transcripts (Fig. 8). Suggesting that these early time gene transcripts up-regulation would be relevant to the manifestation of disease phenotypes (histopathology) at later time points. Two transcripts that are unique to cotton rat (TR453762 and TR53487) were among the top up-regulated host genes at 4 days p.i. Other up-regulated host genes include Interferon-induced GTP-binding protein Mx2 (MX2), 2′-5′-oligoadenylate synthase-like protein 2 (OASL2), Antigen peptide transporter 1 (TAP1), and several Interferon-induced proteins (IFIT1, IF44L). The Mx genes are important immune genes that help mammals fight many RNA and DNA viruses, including HIV, measles, and flu. Previous studies have shown that Mx2 mRNAs was strongly induced in the lungs of RSV-infected animals: the peak expression of the cotton rat Mx proteins occurred on day 4, and declined by day 7 when no virus could be isolated from the lungs^25^. We observed a similar expression profile for the cotton rat Mx2 gene. On 4 days p.i., Mx2 is one of the top up-regulated genes, while at 6 days p.i., the expression level of Mx2 was not significant compare to controls. The CXCL9 gene, a T cell chemoattractant, was strongly induced at 6 days p.i., an indication of enhanced T-cell involvement after RSV infection. A similar observation was previously reported for lung tissue in mice infected with RSV, which were quantified using quantitative qRT-PCR^26^. Antigen peptide transporter 1 (TAP1) was up-regulated at 6 days p.i. TAP1 is involved in the transport of antigens from the cytoplasm to the endoplasmic reticulum for association with MHC class I molecules. Several Guanylate-binding proteins (GBP5, GBP6, and GBP7) are up-regulated 6 days p.i., the GBPs were used extensively to promote understanding of interferon-induced gene transcription and as markers of interferon responsiveness. GBPs exhibit properties like nucleotide-dependent oligomerization and concentration-dependent GTPase activity are associated with functions involve protection against intracellular pathogens^27^. The genes for host cellular defense responses to viral infection are broadly unregulated at 4 days and 6 days p.i. Similarly, the host cellular components involved in RNA capping, immune system process and response to antigen that are highly represented in the up-regulated genes after RSV infection (Table 2). Transcriptomic analyses provide powerful tools to investigate the molecular basis of host responses to viral infection. In a human cohort study, Piters *et al*., analyzed whole-blood transcriptome in children with RSV infection. Most of the children with RSV infection showed overexpression of Interferons (IFN) related genes, independent of the nasopharyngeal microbiota cluster^28^. Similar to the human transcriptional expression, interferon-related genes are upregulated in the cotton rat lung tissue samples upon RSV infection but further studies are needed to confirm these findings.

The three potential major limitations of this study is that we only used the lung tissue sample to generate cotton rat transcriptome; for a comprehensive transcriptome for an animal model, it is better to use transcripts from different tissues and organs of cotton rat. Secondly, a mock (buffer) group should have been included as an background control, so to assess the host transcriptional response to both UV-inactivated and live RSV infections. Thirdly, we only looked at the day 4 and 6 p.i.; however, validation by qRT-PCR shows, these genes are up-regulated and peaked at 24 hrs. In spite of these limitations, our study has considerable strengths. This is the first cotton rat transcriptome study to date and also the first study that shows changes in gene expression in lung tissues, the actual site of viral replication, unlike many previous published studies showing gene expression changes upon RSV infection of bulk blood samples^28^. Taken together, our study provides detailed insights into the lung transcriptome that has allowed us to profile host immune response to active RSV infection. The availability of this additional transcriptome reference sets from RSV infected cotton rat studies will serve as valuable resources to the research community, for further characterization of essential pathways involved in antiviral defense system, adaptive immune mechanisms, and clearance of RSV infections. Findings from this study will especially complement clinical trials in human as cotton rat has been the best pre clinical model for any RSV vaccine studes.

## Methods

### Viral infection

Inbred cotton rats were maintained at Sigmovir Biosystems Inc., Rockville, MD and housed in large polycarbonate cages and fed a diet of rodent chow and water. The cotton rat colony was maintained free of paramyxoviruses, including RSV, and rodent viruses. The animals were 4–6 weeks old and weighed ~100 g at the start of the experiment. All animal experimentation procedures were done following NIH and USDA guidelines and were approved by the Sigmovir Biosystems Inc. Institutional Animals Care and Use Committee.

The prototype Long strain of RSV (RSV A/Long, ATCC Cat. # VR-26, stock titer: 5 × 10^7^ PFU/ml) was propagated in HEp-2 cells and infectious titer determined as previously described^29^. Cotton rats were inoculated intranasally under isoflurane anesthesia with 100 μl of RSV suspension containing the indicated 10^5^ pfu. UV-inactivated RSV at equivalent doses was used as controls. Animals were sacrificed by carbon dioxide inhalation at the indicated intervals thereafter. Immediately after dissecting the whole lungs out, we excised lingular lobe of and snap frozen in liquid nitrogen for RNA extraction and for transcriptome and validation RT-qPCR assay, left lobe for virus titration, and right lobe were inflated with 10% buffered formalin for histology.

### Lung histopathology

Lungs (right lobe) were dissected and inflated with 10% neutral buffered formalin and immersed in formalin for fixation. Lungs were embedded in paraffin blocks, sectioned, and stained with hematoxylin and eosin (H&E). Slides were read blindly, and examined for 4 parameters of pulmonary inflammation: peribronchiolitis, perivasculitis, interstitial pneumonia, and alveolitis^19,20^.

### RNA extraction and cDNA library preparation

Cotton rat lung tissue was homogenized using a bead beater, and total RNA was extracted using Qiagen’s miRNeasy mini kit, with on column DNase digestion as recommend by the manufacturer’s instructions (Cat: 74104). The RNA quality was measured using an Agilent 2100 Bioanalyzer (Agilent Technologies, CA, USA). We used 4 μg of “total RNA” from each sample to make cDNA libraries; the ribosomal RNA was removed using Ribo-Zero rRNA Removal Kit (Epicentre), and cDNA libraries were made using the TruSeq RNA Library Preparation Kit v2 (Epicentre). Each sample was labelled with a unique barcode sequence to enable multiplexing of all samples across one lane. Paired-end sequencing with 2 × 150 bp read module was performed on an Illumina NextSeq500 instrument. The resulting sequence was parsed into individual libraries by barcode, and then preprocessed to eliminate adaptor sequences and low quality and short reads using a minima Phred quality of 33.

### Transcriptome assembly

The sequencing reads were processed to remove the low quality reads before assembly. The Illumina adapters were removed using Trimmomatic (version 0.36)^12^ for quality trimming of the reads using a sliding window (4 bp with a minimal Phred quality of 33); reads shorter that 80 bp were discarded. Paired-end and singletons reads which passed the desired quality threshold were retained. About 71926634 paired-end reads were sampled for *de novo* transcriptome assembly using the Trinity (version 2.0.6) software package^7,30^, with a default parameter set (–glue_factor 0.01–glue_factor 0.01–min_iso_ratio 0.1–min_iso_ratio 0.1). After assembly, the transcripts with a length of <200 nucleotides were removed from the assembly. General transcriptome statistics, including maximal transcript length, mean transcript length and N50, of the resulting transcripts were listed in Table 1. This transcriptome assembly data will be deposited in BioProject upon acceptance of the manuscript for publication.

The RSEM package was used for quantifying gene and isoform abundances from paired-end RNA-Seq data^13^. The RSEM does not require a reference genome, in combination with a *de novo* transcriptome assembler; RSEM enables accurate transcript quantification per sample. The transcriptome FPKM (Fragments Per Kilobase of transcript per Million mapped reads) was calculated using the RSEM package with Bowtie2 aligner and a cut off of FPKM > 1 was used to filter the low quality assembled transcripts.

### Transcriptome characterization

The Trinotate annotation pipeline (Version V3.0) was used to annotate cotton rat transcripts. Peptide coding regions were found through transdecoder and BLASTp v 2.2.28 (e-value cutoff of 1e-5) was used to find sequence homology to UniProt/SwissProt. HMMER3 web server was used to identify conserved protein domains^31^. SignalP 4.0 was used to predict signal peptides from transmembrane regions^32^. The transmembrane regions were predicted with TMHMM-2.0c and potential signal peptides identified with SignalP. Moreover, homology searches were performed using BLASTx against NCBI non-redundant (nr) protein database with an e-value cutoff of 1e-5. Functional annotation analysis was conducted by assigning Molecular Function, Biological Process and Cellular Component Gene Ontology annotations to transcripts with BLASTx.

### Differential gene expression and GO terms

For each sample the RNA-Seq reads were mapped to the *de novo* assembled cotton rat transcripts using RSEM^13^ to obtain overall transcript expression values. Differential transcript expression was performed by comparing three biological replicates of group with the corresponding control group samples. The mapped reads counts were performed with RSEM based on the edgeR package^13,21^ with a dispersion parameter of 0.4, which is recommended for analysis without replicates. Transcripts with an FDR < 0.05 were treated as differentially expressed. The lists of differentially expressed genes for each tissue were analyzed for enrichment of Gene Ontology categories using BLAST2GO, and terms were deemed significant when FDR < 0.05. We used goseq package in Bioconductor to detect differentially abundant GO terms^33^. For clustering of protein sequences, we used the CD-HIT package to perform similarity-based clustering at 90% sequence similarity.

### Quantitative real-time PCR analysis

RNA extracted from the lung tissue was used for qRT-PCR assays. The cDNA was prepared using QuantiTect Reverse transcription kit (Qiagen) that uses SYBR Green. Each cDNA reaction was prepared from 1 μg of RNA, diluted to 100 μl of the final volume and 3 μl of cDNA was subsequently used for each PCR reaction. Real-time PCR primers were designed for each transcript (TR453762, TR529629, and TR5333) based on *de novo* assembled transcripts. The primer sequences used for the qRT-PCR assay are listed in Table 4. The PCR amplicons were gel-purified and sequenced to confirm the specificity of primers. Levels of each cytokine mRNA was normalized by comparison to expression of the β-actin housekeeping gene and reported as fold induction over uninfected lungs.

**Table 4:** Nucleotide sequence of primers used for qRT-PCR.

Primer name	Nucleotide sequence (5′ to 3′)
TR453762 F	GCAGTCTCTGGGGATCTGAA
TR453762 R	TGGCTGTGTAAGTGTTGAAAGG
TR529629 F	AGGCAGGAAATTGGATAGGAA
TR529629 R	GACTAGCCTTTGAGCATATTGTGAG
TR5333 F	ATGTGTGTGCAGGAACAGGA
TR5333 R	CATCTCTTGAGGAAGGGAGTAA

## Electronic supplementary material

Supplementary table 1
